# Perceived importance of substance use prevention in juvenile justice: a multi-level analysis

**DOI:** 10.1186/s40352-018-0070-9

**Published:** 2018-05-15

**Authors:** Jessica M. Sales, Gail Wasserman, Katherine S. Elkington, Wayne Lehman, Sheena Gardner, Larkin McReynolds, Tisha Wiley, Hannah Knudsen

**Affiliations:** 1 Department of Behavioral Sciences and Health Education, Emory University Rollins School of Public Health, 1518 Clifton Road, Room 570, Atlanta, GA 30322 USA; 2 Columbia University, 1051 Riverside Drive, Unit 78, New York City, NY 10032 USA; 3 Texas Christian University, 3034 Sandage Avenue, Fort Worth, Texas 76129 USA; 4 Mississippi State University, 1 Research Blvd., Suite 103, Starkville, MS 39759 USA; 5 National Institute on Drug Abuse, Frankfort, USA; 6 University of Kentucky, 845 Angliana Avenue, Room 204, Lexington, KY 40508 United States

**Keywords:** Juvenile justice, Community supervision, Substance use prevention

## Abstract

**Background:**

Youth under juvenile justice (JJ) supervision are at high-risk of adverse outcomes from substance use, making prevention important. Few studies have examined prevention-related attitudes of JJ employees, yet such attitudes may be important for implementing prevention programs. Attitudes toward prevention may reflect individual characteristics and organizational contexts.

**Methods:**

Mixed effects regression was used to analyze data from 492 employees in 36 sites participating in the Juvenile Justice—Translational Research on Interventions for Adolescents in the Legal System (JJ-TRIALS) cooperative agreement. JJ employees’ perceived importance of substance use prevention was measured. Staff-level variables included attitudes, job type, and demographic characteristics. Site-level variables focused on use of evidence-based screening tools, prevention programs, and drug testing.

**Results:**

On average, JJ employees rated substance use prevention as highly important (mean = 45.9, out of 50). JJ employees generally agreed that preventing substance use was part of their agency’s responsibility (mean = 3.8 on scale ranging from 1 to 5). At the site level, 72.2% used an evidence-based screening tool, 22.2% used one or more evidence-based prevention program, and 47.2% used drug testing. Reported importance of prevention was positively associated with site-level use of screening tools and drug testing as well as staff-level attitudes regarding prevention being consistent with the agency’s mission.

**Conclusions:**

The associations between screening and prevention attitudes suggest that commitment to identifying youth needs may result in greater openness to preventing substance use. Future efforts to implement substance use prevention within JJ agencies charged with supervising youth in the community may benefit from highlighting the fit between prevention and the agency’s mission.

## Background

Substance use is prevalent among American youth, and its associated negative consequences render adolescent substance use a significant public health problem. Data from the 2015 Monitoring the Future study indicated that 15.8% of youth reported past-month illicit drug use and 19.9% reported consuming alcohol in the previous month Johnston et al. (2017). Adolescent substance use is concerning for its short-term negative consequences, including its associations with risky sexual behaviors (Ritchwood et al. 2016), reduced academic achievement (Arthur et al. 2015), and suicidal behaviors (Gart and Kelly 2015). Although trajectories of use over time are dynamic and not uniform (Brooks-Russell et al. 2015; Lamont et al. 2014), substance use during adolescence increases the likelihood of the development of substance use disorder (SUD) during adolescence (Marti et al. 2010; Winters and Lee 2008) and adulthood (Englund et al. 2008; McCabe et al. 2016; Stone et al. 2012; Swift et al. 2008).

Youths with juvenile justice (JJ) system contact have rates of SUD that are substantially higher than their non-justice-involved peers. Prior studies have found between 20% and 51% of JJ youth report a SUD (Teplin et al. 2006; Wasserman et al. 2010) compared to 11% in the general population (Merikangas et al. 2010), which if unaddressed and untreated can significantly complicate their justice involvement. Among justice involved youth, substance use has been associated with a range of health and behavioral health adverse conditions, such as trauma exposure (especially assaultive violence), suicidal behavior (Nolen et al. 2008; Wasserman and McReynolds 2006; Wasserman and McReynolds 2011), elevated sexual risk behaviors and STIs (Teplin et al. 2003; Elkington et al. 2010) and school dropout. Undetected and untreated, justice-involved substance-using youths are particularly likely to recidivate (Hoeve et al. 2013c; Schubert et al. 2011), and to continue offending into adulthood (Hoeve et al. 2013a), escalating the public health consequences of their behavior. Indeed, rates of SUD increase significantly as youth penetrate the justice system, from community supervision to secure care (Wasserman et al. 2010). On the other hand, when justice-involved youths are diverted to behavioral health services, their recidivism risk is decreased (Cuellar et al. 2006; Hoeve et al. 2013b), along with their behavioral health needs.

Substance use prevention, both primary and secondary prevention efforts, for justice-involved youth is likely critically important to disrupt pathways to multiple potential adverse physical, mental and behavioral outcomes. Substance use prevention programs aim to delay or prevent onset of use among those who have never used illegal substances (i.e., primary prevention), as well as to reduce escalation in use among those who have already begun using (i.e., secondary prevention). Effective prevention programs work to increase and enhance protective factors while decreasing risk factors that have been linked to substance use. For example, a review of evidence-based interventions for preventing substance use and abuse highlights exemplary prevention programs for implementation at the individual, group, family, school or community level (see Griffin and Botvin 2010 for details), including programing that has been developed specifically for justice-involved youth and their families (e.g., Mutlisystemic Therapy (MST); Multidimensional Family Therapy (MDFT)).

In recent years, many states across the U.S. have overhauled their JJ systems in order to reduce the number of youth in detention or locked facilities and decrease recidivism. To accomplish this, justice reform has prioritized addressing underlying issues contributing to youth involvement in the justice system, such as substance use, through connection to community-located services. The implications of this shift increases the number of youth under community supervision and thereby allows juvenile probationers to live in communities with access to drugs and alcohol. However, if effectively linked to and engaged in evidenced-based community-located substance use programs (e.g., gold-standard family treatment programs that leverage family support), particularly when identified early before substance use progresses, youth outcomes should improve and future involvement with the justice system should decline. Within the JJ system, the focus of community supervision agencies and their staff is to reduce recidivism, in part by linking youth to needed services, including substance use prevention programs. Yet, relatively little is known about the attitudes and practices pertaining to substance use prevention among the JJ staff and agencies charged with community supervision of juvenile probationers.

JJ agencies, and particularly community supervision staff, are ideally positioned to play a vital role in substance use prevention among youth with whom they come into contact, either by active linkage to community-located substance use prevention programs or through implementation of substance use prevention programs within their agency. The Gateway Provider Model (GPM) acknowledges that the “gateway provider” into substance use related services is not always a parent or behavioral health provider, but often is another system provider (child welfare, juvenile justice, or education). The GPM considers the interdependence of the gateway provider, organizational contextual factors, *and* the youth/family when understanding or determining service use/provision. After identifying need, gateway providers make decisions based on their services knowledge and attitudes toward treatment and the availability of services within an organizational context that supports or hinders this process (Stiffman et al. 2004). According to the GPM, the extent to which substance use prevention efforts are enacted within JJ agencies is likely dependent upon multiple factors ranging from individual characteristics of JJ employees to organizational policies and practices supportive of prevention. Based on research from adult corrections settings, including research focused on parole staff, individual staff characteristics such as age (Stohr et al. 2000), race (Cullen et al. 1989), and gender (Aarons and Sawitzky 2006) are associated with workplace behavior and can contribute to or impede the implementation of evidence-basedpractices (EBPs) within an organization. Recent research in community-based treatment agencies has highlighted the importance of certain individual and organizational characteristics, including administrator and staff attitudes (Knudsen et al. 2005; Liddle et al. 2002; Schmidt and Taylor 2002), organizational climate (Aarons and Sawitzky 2006; Glisson 2002) and structure (Knudsen et al. 2006; Roman and Johnson 2002) which are conducive to the adoption of EBP’s. Notably, these studies have been almost entirely limited to treatment-focused EBPs in community-based mental health or treatment settings. Few studies have examined the aforementioned individual and organizational characteristics related to substance use prevention in JJ agencies, which is a concerning gap given the critical role JJ staff play in linking probated youth to needed community services.

JJ reform prioritizes keeping youth in communities whenever possible to better address root causes of justice-involvement, such as substance use, as a means to reduce recidivism and improve youth outcomes. The primary focus of community supervision agencies and their staff is to reduce recidivism, in part by linking youth to needed services, including substance use prevention programs. Following from the GPM, prevention-related attitudes of JJ employees are critical for implementing prevention programs in JJ settings. Moreover, JJ employee attitudes toward substance use prevention may reflect individual characteristics and organizational contexts. Yet, relatively little is known about the attitudes related to substance use prevention and the specific prevention practices employed in substance use prevention programs (e.g., strengthening youth’s drug refusal skills; strengthening family skills) of the JJ staff and agencies charged with youth under community supervision. Thus, the aim of this paper is to describe attitudes towards substance use prevention among JJ staff in community supervision agencies, and to estimate a multi-level model of perceived importance of substance use prevention that integrates empirically derived individual (i.e., age, gender, race/ethnicity, job type, perception of prevention as part of agency mission) and agency-level characteristics (i.e., use of evidence-based substance use screening, routine drug testing, and prevention programs).

## Methods

This study draws upon data collected as part of the *Juvenile Justice-Translating Research Interventions for Adolescents in the Legal System* (JJ-TRIALS) cooperative research, which is supported by the National Institute on Drug Abuse (NIDA). JJ-TRIALS consists of six research centers (Columbia University, Emory University, Mississippi State University, Temple University, Texas Christian University, and University of Kentucky) and a coordinating center (Chestnut Health Systems). As part of a larger study to improve the delivery of evidence-based substance use services for justice-involved youth under community supervision (i.e., probation or youth drug court), data were collected from employees working within 36 JJ sites, which were located in Florida, Georgia, Kentucky, Mississippi, New York, Pennsylvania, and Texas. Each research center recruited its own JJ sites in collaboration with state JJ agencies based on six eligibility criteria. JJ sites were required to (1) be able and willing to provide youth service records, (2) have youth who were under community supervision, (3) have access to a behavioral treatment provider if on-site treatment was not available (notably, most sites did not directly deliver treatment), (4) have an average monthly case flow of at least 10 youths, (5) employ at least 10 staff within the site, and (6) identify a senior JJ staff person who was willing to act as a site liaison during the project (Knight et al. 2016).

### Data collection

All employees working within the 36 JJ sites were invited to participate in a baseline staff survey, with data collection beginning in August 2015 and ending March 2016. Recruitment occurred either through a face-to-face orientation in which the JJ-TRIALS main protocol was described or through email correspondence or telephone calls conducted by research staff. Employees were assured that participation was voluntary and would not impact their employment within the JJ site; informed consent was obtained. The consent process included participation in the overarching protocol as well as the baseline survey; of the 760 JJ staff who were eligible to participate in the JJ-TRIALS protocol, 595 consented to be in the study (78.3%). Surveys were administered by either a Qualtrics® web-based survey that used individualized invitation links or a paper survey. Surveys were received from 492 JJ employees (83.7% response rate among consented staff).

In addition to the staff surveys, a member of agency leadership within each of the 36 sites was invited to complete a questionnaire consisting of measures that were focused on the site-level. This questionnaire captured information regarding the range of substance use screening, assessment, and treatment services as well as mental health and HIV-related services that were delivered within the site. Data were obtained from all 36 sites.

### Measures

#### Employee-level measures

Variables measured at the employee-level via the survey included the dependent variable of perceived importance of substance use prevention as well as the independent variables of the perception that substance use prevention was one of the agency’s responsibilities, job type, job setting, and demographic characteristics. The measure of *perceived importance of substance use prevention* consisted of 6 items about prevention for justice-involved youth under community supervision; item wording appears in Table 1. Respondents were asked to rate the importance of each item, with response options ranging from 1 = not important to 5 = very important. The mean of the responses was calculated and multiplied by 10 (alpha = .94). A single item asked respondents to rate their agreement with the statement, “My agency’s responsibilities include prevention of youth substance use,” with responses ranging from 1 = strongly disagree to 5 = strongly agree. Responses were grand mean-centered for the multilevel regression model. Job type categorized respondents into probation officers (=1) and all others (=0; e.g., director, supervisor, support staff). Respondents also indicated whether they worked in a behavioral health unit within the JJ agency (=1) or non-behavioral health unit (=0). Demographic characteristics included *age* in years (which was grand mean-centered in multilevel regression model), *gender* (1 = female, 0 = male), and a measure of *race/ethnicity* that drew upon measures of Latino ethnicity and race. Based on small cell sizes for certain groups, respondents were coded as non-Hispanic whites (reference group), non-Hispanic African Americans, or all others.

**Table 1:** Descriptive statistics for employees working in juvenile justice agencies

	Mean (SD) or % (N)	Available N
Perceived importance of substance use prevention		
Strengthening youth’s anti drug use attitudes, beliefs, and norms	4.55 (0.62)	479
Strengthening youth’s life skills	4.65 (0.55)	479
Strengthening youth’s drug refusal skills	4.60 (0.61)	475
Strengthening family skills	4.62 (0.56)	475
Strengthening caring relationships with people in the youth’s network who do not endorse substance use	4.58 (0.59)	479
Ensuring that prevention strategies are developmentally, culturally, and linguistically appropriate for the populations	4.51 (0.68)	478
Mean scale of perceived importance of substance use prevention	45.87 (5.31)	473
Substance use prevention as part of agency’s responsibilities	3.85 (0.85)	485
Job type		485
Probation officer	60.6% (294)	
All other job types	39.4% (191)	
Unit type		474
Behavioral health unit within juvenile justice agency	12.5% (59)	
Non-behavioral health unit	87.6% (415)	
Age in years	41.53 (9.68)	477
Gender		
Female	59.1% (290)	491
Male	40.9% (201)	
Race/ethnicity		477
Non-Hispanic white	64.2% (306)	
Non-Hispanic African American	23.7% (113)	
All others	12.2% (58)	

*Notes.* Percentages may not sum to 100% due to rounding. Responses to the importance of substance use prevention items ranged from 1 = not important to 5 = very important; for the scale score, the mean was multiplied by 10

#### Site-level measures

Three site-level measures were included, which were obtained via questionnaires completed by a leader within each site. The site-level questionnaire was modeled upon a survey fielded with a nationally representative sample of JJ system-based community supervision agencies (Scott, CK, Dennis, ML, Lurigio, AJ: Juvenile justice system-based community supervision agencies: Results of a national survey on behavioral health screening, assessment, referral, prevention, and treatment practices, submitted). First, a measure of *evidence-based screening* was constructed based on responses to a question that asked, “In your agency, which of the following instruments do your staff members currently use to screen for substance use, HIV and mental health problems?” Response options included a list of 44 “name brand” evidence-based screening tools, an option for no screening, and an option for a locally developed screening tool. The measure of evidence-based screening was coded 1 if the site indicated using at least one of the 44 evidence-based screening tools and 0 if none of the evidence-based screening tools were selected. Second, *use of drug testing* during screening was a dichotomous measure, coded 1 if sites indicated drug tests were routinely collected as part of the screening process and 0 if drug tests were not routinely collected. Finally, *use of evidence-based prevention* was based on responses to the question, “Which substance use prevention program(s) does your agency currently provide?” Response options included 66 “name brand” evidence-based substance use prevention programs. Sites were coded into one of three mutually exclusive groups: those that endorsed at least one of the evidence-based programs, those that used a locally developed prevention program, and those that did not provide substance use prevention (reference).

#### Analysis

All analyses were conducted in *Stata 13.1* (StataCorp, College Station, TX). Descriptive statistics were calculated for all variables. Rates of missing data for staff-level variables ranged from 0.2% (for gender, *n* = 1) to 3.7% (for the dependent variable and job setting, *n* = 19). To reduce the risk associated with complete case analysis, (Allison 2009), multiple imputation by chained equations (MICE) was used (White et al. 2011). The specification of the “mi impute chained” command included all independent (staff-level and site-level) variables and the dependent variable. Fifteen datasets were generated.

To account for nesting of employees within sites and to model the site-level variables, mixed effects regression was implemented using a random intercepts model. The number of employees within the 36 sites ranged from 2 to 50. Two models were estimated. Model 1 only included employee-level variables, with a *p*-value of .05 indicating statistical significance (two-sided test). Then, the three site-level variables were added to the model.

## Results

### Descriptive statistics

Descriptive statistics for attitudes and characteristics of JJ staff are presented in Table 1. On average, JJ employees reported very strong support for the importance of substance use prevention, with a mean (45.9) that neared the maximum of the scale (maximum = 50). JJ staff generally agreed that preventing substance use was part of their agency’s responsibilities (mean = 3.8 on scale ranging from 1 to 5).

At the site-level, 72.2% of sites (*n* = 26 of 36 sites) reported use of at least one evidence-based screening instrument, and 47.2% of sites (*n* = 17) routinely used drug testing as part of the screening process. The most commonly endorsed screening instrument was the Massachusetts Youth Screening Instrument-2 (MAYSI-2; *n* = 20). Six sites reported use of the Substance Abuse Subtle Screening Inventory (SASSI), 5 used the Youth Self-Report, 5 indicated use of the CRAFFT, 2 reported using the Global Appraisal of Individual Needs-Quick Version 3 (GAIN-Q3), and 1 site used the Child and Adolescent Needs & Strengths (CANS). Fifteen sites (41.7%) reported using one screening instrument, 9 sites (25.0%) used two screening instruments, and 2 sites (5.6%) used three screening instruments.

Regarding substance use prevention, 8 sites (22.2%) reported using one or more of the evidence-based prevention programs, 9 sites (25.0%) indicated they provided locally developed or non-evidence-based prevention, and 19 sites (52.8%) did not provide substance use prevention. Of the 8 sites offering evidence-based substance use prevention, 7 sites reported use of just one program and 1 site indicated provision of 6 prevention programs. The specific prevention programs provided were Life Skills Training (*n* = 4), Active Parenting Now (n = 2), Big Brothers Big Sisters of America Mentoring (*n* = 1), Communities that Care (n = 1), Drug Abuse Resistance Education (DARE; n = 1), Parenting with Love and Limits (n = 1), Project SUCCESS (Schools Using Coordinated Community Efforts to Strengthen Students; n = 1), Safe Dates (n = 1), and Strengthening Families Program for Parents and Youth 10–14 (n = 1).

### Mixed effects regression models

Table 2 presents two models of perceived importance of substance use prevention. Model 1 only includes employee-level measures. Four employee variables were significantly associated with the perceived importance of substance use prevention. There was a positive association between the perception that prevention was part of the agency’s responsibilities and perceived importance of prevention. Female staff rated the importance of substance use prevention greater than did men. Compared to white JJ employees, African Americans reported greater importance of substance use prevention. Older employees rated perceived importance of substance use prevention greater than younger employees.

**Table 2:** Mixed effects regression model of perceived importance of substance use prevention

	Model 1 Unstandardized coefficient (95% CI)	Model 2 Unstandardized coefficient (95% CI)
*Employee-level variables*		
Substance use prevention as part of agency’s responsibilities (grand mean-centered)	1.104*** (0.564, 1.644)	1.074*** (0.550, 1.599)
Probation officer (vs. all other job types)	−0.813 (− 1.823, 0.196)	− 0.415 (− 1.396, 0.566)
Works in behavioral health unit (vs. non-behavioral health unit)	0.391 (− 1.006, 1.789)	0.337 (− 1.028, 1.702)
Age in years (grand mean-centered)	0.071** (0.019, 0.122)	0.078 (0.028, 0.128)
Female (vs. male)	1.466** (0.526, 2.407)	1.443** (0.529, 2.357)
Race/ethnicity		
Non-Hispanic white	Reference	Reference
Non-Hispanic African American	1.313* (0.115, 2.511)	1.345* (0.215, 2.474)
All others	0.304 (− 1.196, 1.804)	0.208 (− 1.236, 1.652)
*Site-level variables*		
Use of at least 1 evidence-based screening instrument (vs. no evidence-based screening instrument)	–	1.905** (0.788, 3.022)
Routine of drug testing during screening (vs. no routine use)	–	1.722*** (0.800, 2.644)
Substance use prevention programming		
Use of at least 1 evidence-based prevention program	–	0.738 (−0.388, 1.865)
Use of a locally developed prevention program	–	0.343 (− 0.819, 1.505)
No substance use prevention program	–	Reference
*Constant*	45.103 (44.042, 46.165)	42.440 (40.994, 43.886)
*Random-Effects Parameters*		
*Variance(constant)*	0.880 (0.406, 1.903)	1.38E^−09^ (5.84E^−13^, 3.25E^− 06^)
*Variance(residual)*	4.988 (4.672, 5.324)	4.902 (4.600, 5.224)

*Notes.* *p < .05, **p < .01, ***p < .001 (two-tailed tests). Results reflect the pooled estimates from 15 imputed datasets (*n* = 492)

Table 2, Model 2 presents a model including the 3 site-level variables. There was a positive correlation between use of evidence-based screening and perceived importance of substance use prevention. Similarly, routine use of drug testing was positively correlated with perceived importance of substance use prevention. However, the provision of evidence-based prevention was not significantly associated with its perceived importance. The employee-level variables that were significant in Model 1 were significant after controlling for the 3 site-level variables.

## Discussion

Substance use prevention, both primary and secondary prevention efforts, for justice-involved youth is likely critically important to disrupt pathways to multiple potential adverse physical, mental and behavioral outcomes, including further involvement with the justice system. Given the important role that community supervision agencies and their staff play in linking probated youth to the prevention and treatment services they need (i.e., their role as gatekeepers), it is imperative to better understand individual- and agency-level characteristics that might better facilitate this process. Previous research on community-based treatment agencies suggests that the implementation of substance use prevention programs in JJ settings is likely impacted by employee and agency characteristics. The aim of this paper was to examine the relationship between these characteristics and attitudes towards substance use prevention among JJ employees, specifically community supervision staff. Overall, JJ community supervision staff perceived substance use prevention as highly important. However, we also found associations between staff perceptions of the importance of substance use prevention and several individual- and agency-level characteristics. Women, African Americans, older staff, and persons who believed that substance use prevention is part of the agency’s responsibility rated substance use prevention as more important. Agency-level characteristics positively associated with staff perceptions of the importance of substance use prevention included agency use of evidence-based screening and routine use of drug testing.

When thinking about JJ community supervision staff as potential gatekeepers to substance use prevention and treatment services, our observed overall high ratings of the importance of substance use prevention by community supervision staff in JJ agencies is encouraging. Though the primary focus of their job is to reduce recidivism, this goal is in part achieved by preventing or reducing youth engagement in substance use, either through linkage to community programs or through the provision of prevention programs within JJ services. Attitudes acknowledging the importance of substance use prevention for youth under their supervision may serve as a key indicator of willingness to implement prevention programs within agencies that supervise probated youth. Notably, in this sample of JJ agencies, relatively few were utilizing a name-brand prevention program, and more than half of the sites did not offer any formal prevention services. For leadership within JJ agencies considering providing substance use prevention services within their setting, the generally strong support for preventing youth substance use among JJ staff suggests that implementing prevention programs would not face philosophical resistance from staff, although there may be other implementation barriers related to other work-related demands placed on staff. With this in mind, organizational changes often are needed to facilitate the adoption and implementation of EBPs, and in probation settings, this is often a long and complex process and typically requires both extensive planning and training (Baer et al. 2007; Simpson 2002). The GPM acknowledges the interdependence of gateway providers and organizational contextual factors. Thus, in addition to positive staff attitudes, research suggests that having motivated and committed leadership, an organizational mission statement and values that promote change in organizational culture, and collaboration between justice agencies and service providers are key components of successful EBP implementation in justice settings (Clawson et al. 2005; Joplin et al. 2004).

Despite the high rating of importance of substance use prevention by JJ staff, there were differences in individual-level staff characteristics associated with perceived importance of substance use prevention suggesting that certain staff value substance use prevention more highly than others. Specifically, demographic characteristics of gender, race/ethnicity and age were all significantly associated with rating of importance, suggesting that agency-wide trainings, especially for younger staff who may be new to their positions, may be useful. Such trainings should highlight the importance of substance use prevention for improving youth outcomes and decreasing their future involvement with the justice system, and explicitly set the expectation that most behaviorally-focused prevention programs have incremental impact on substance use behavior observed in the aggregate across youth, which may not be readily observable to staff during their interactions with individual youth. In addition to trainings, agencies providing substance use prevention services should consider developing or strengthening mechanisms to share aggregate findings across youth so that staff can recognize incremental, positive gains attributed to prevention services. Taking such proactive steps to enhance JJ community supervision staffs’ attitudes regarding substance use prevention services could positively influence their decisions to connect identified youth in need to community-located substance use prevention services.

Similar to the literature from community-based treatment agencies highlighting the importance of certain organizational characteristics, including organizational climate (Aarons and Sawitzky 2006; Glisson 2002) and structure (Knudsen et al. 2006; Roman and Johnson 2002) conducive to the adoption of EBPs, our findings highlight JJ agency-level factors are associated with staff attitudes about substance use prevention. The use of evidence-based substance use screening and routine use of drug testing among JJ agencies were predictive of staff’s rating of importance of substance use prevention. Thus, when JJ agencies adopt practices that show their strong commitment to identifying the substance use behaviors of youth under their supervision this is positively related to the attitudes their staff hold pertaining to the value of substance use prevention.

Our finding that agency-level factors are important in staff attitudes related to prevention is reinforced through our finding that staff who believed substance use prevention is part of the agency’s responsibility rated substance use prevention as more important. Staff-level beliefs about the agency’s responsibility pertaining to substance use prevention may be directly influenced through agency-level characteristics that demonstrate the organization’s values and commitment to the substance use related needs of youth served by their agency. Thus, JJ community supervision agencies should highlight and communicate to their staff how substance use prevention aligns with the agency’s mission of reducing recidivism to further strengthen the attitudes towards prevention among their staff.

Several limitations about this study should be noted. The study sample of the 36 JJ sites was non-random, representing 7 states. Though there was considerable structural and demographic diversity across the states in this sample, we do not know the degree to which results generalize to other states or to other counties or agencies within the 7 states represented. Findings from a national survey of JJ community supervision (Scott et al., submitted) agencies suggest that the JJ-TRIALS sites are more focused on substance use screening, drug testing, and evidence-based prevention than most agencies in the nation. Nationally, only half of agencies have adopted at least one evidence-based screener, about 25% routinely use drug testing as part of screening, and very few agencies (8%) offer one or more evidence-based prevention programs. In contrast, among the 36 JJ-TRIALS sites, 72% used evidence-based screening, 47% routinely used drug testing, and 22% offered evidence-based prevention programming.

Additional limitations are related to the study design and measures used. The survey was cross-sectionally assessed at baseline in terms of the larger study design. Thus, causal inferences cannot be made and we provide no data on stability of responses or changes in attitudes across time. The outcome variable was a self-report measures of the perceived importance of substance use prevention and did not include behavioral measures of prevention services (e.g., making referrals to prevention services, individual-level delivery of prevention services). In addition, the overall mean of the dependent measure was near the maximum scale score, indicating very high agreement on the importance of prevention services and potential social bias of our assessment. There was also high agreement that substance abuse prevention was part of their agencies’ responsibilities. This high agreement and low variance in the dependent measure means less predictable variance which can limit the associations with predictors. The measures used also did not differentiate between different levels or types of prevention services such as primary (preventing initiation) or secondary prevention (preventing escalation).

## Conclusions

In spite of these limitations, this study has several important strengths including a large and diverse sample of JJ programs located in 7 different states to examine staff and organizational correlates of perceived importance of prevention services, making it one of the largest studies examining attitudes toward prevention in JJ agencies. Further, the study fills important gaps in the literature related to attitudes of community supervision staff working within the JJ system, which is valuable given their role as gatekeepers to linking youth to needed substance use prevention and treatment services in the community. Results are encouraging in that there is very strong support among community supervision staff for substance use prevention services and widespread agreement that prevention services are part of agency responsibilities. Both may be important indicators of willingness on the part of JJ staff to implement prevention programs within JJ settings serving the needs of youth under community supervision. JJ agencies may further strengthen their staff’s attitudes towards substance use prevention by communicating how prevention aligns with the agency’s mission and the larger goals of justice reform, community diversion and reducing recidivism among youth.

## Abbreviations

CODs: Co-occurring disorders
EBPs: Evidence-based practices
JJ: Juvenile Justice
JJ-TRIALS: Juvenile Justice—Translational Research on Interventions for Adolescents in the Legal System
MH: Mental Health
MICE: Multiple imputation by chained equations
NIDA: National Institute on Drug Abuse
SU: Substance Use
SUD: Substance use disorder
